# Oxathiapiprolin-based fungicides provide enhanced control of tomato late blight induced by mefenoxam-insensitive *Phytophthora infestans*

**DOI:** 10.1371/journal.pone.0204523

**Published:** 2018-09-27

**Authors:** Yigal Cohen, Avia Evgenia Rubin, Mariana Galperin

**Affiliations:** The Mina & Everard Goodman Faculty of Life Sciences, Bar-Ilan University, Ramat-Gan, Israel; Agriculture and Agri-Food Canada, CANADA

## Abstract

Oxathiapiprolin is a new fungicide with extremely high efficacy against oomycete plant pathogens. Solo components oxathiapiprolin (OXPT), chlorothalonil (CHT), azoxystrobin (AZ), mandipropamid (MPD), and mefenoxam (MFX) were compared with each other and with four oxathiapiprolin pre-packed fungicidal mixtures, OXPT+CHT 1+66.7, OXPT+AZ 1+10.3, OXPT+MPD 1+8.3, and OXPT+MFX 1+3 (weight active ingredient ratio), for control efficacy of late blight induced by MFX-insensitive *Phytophthora infestans* strains in tomato in growth chambers and the field. Mixtures performed better than all partner fungicides alone, except OXPT. Of the four mixtures, OXPT+MFX outperformed, with the highest preventive, curative, translaminar, and systemic efficacies. In the field, OXPT+MFX was superior to other fungicides in controlling late blight epidemics induced by MFX-insensitive isolates. Its deployment in the field will combat the dominating MFX-insensitive isolates, reduce the selection pressure imposed on *P*. *infestans* and delay the buildup of subpopulations resistant to oxathiapiprolin.

## Introduction

Oxathiapiprolin (OXPT) is a new piperidinyl thiazole isoxazoline fungicide (FRAC code U15) discovered and developed by DuPont [1, 2]. Oxathiapiprolin has an extremely high activity against a range of plant pathogenic oomycetes, including *Phytophthora nicotianae* [3], *Pseudoperonospora cubensis* [4], *P*. *capsici* [5], *P*. *infestans* [6], *Peronospora belbahrii* [7], *P*. *cinnamomi* [8], *P*. *parasitica*, and *P*. *citrophthora* [9]. The compound is not active against *Pythium* species [1].

Oxathiapiprolin acts at multiple stages of the pathogen’s asexual life cycle at extremely low concentrations. Preventatively, it inhibits zoospore release, zoospore motility and sporangia germination. Curatively, it stops mycelial growth within the host plant before visible lesions occur, offering protection at one and two days post-infection. It stops mycelial growth and inhibits further lesion expansion, and inhibits spore production. It phenotypically shows translaminar and acropetally systemic movement, protecting treated leaves and new leaves as they emerge and grow [2, 4, 10].

Oxathiapiprolin solo was evaluated in the field for control of the major oomycete diseases of grapes, potatoes, and vegetables. It demonstrated outstanding control of potato late blight, grape downy mildew, cucumber downy mildew and crown and root rot of peppers [2, 10]. Soil-directed applications of OXPT alone or in alternation with mefenoxam, effectively reduced black shank of tobacco [11].

The molecular target of OXPT is the oxysterol binding protein [OSBP; [2, 12]], a member of the OSBP-related protein (ORPs) family of lipid transfer proteins (LTPs). They constitute a family of sterol and phosphoinositide binding and transfer proteins in eukaryotes, conserved from yeast to humans. The lipid-binding proteins were implicated in many cellular processes related to oxysterol, including signaling, vesicular trafficking, lipid metabolism, and non-vesicular sterol transport [13]. Oxysterol-binding protein (OSBP) localizes to endoplasmic reticulum-Golgi contact sites, where it transports cholesterol and phosphatidylinositol-4-phosphate and activates lipid transport and biosynthetic activities [14].

The target protein of OXPT in *P*. *capsici* genome has been annotated, but the function of this protein in *P*. *capsici* or any other oomycete remains unknown [15]. The data presented by Andreasii et al. [12], Miao et al. [10, 15] and Pasteris et al. [2] indicate that OXPT is a high-risk fungicide that requires careful use in the field to avoid development of resistant mutant isolates. Resistance against OXPT was induced in *P*. *capsici* by UV irradiation [12] and by fungicide adaptation [15]. Bittner and Mila [16] reported on the production of *P*. *nicotianae* isolates resistant to OXPT by using UV light mutagenesis and mycelial adaptation.

According to FRAC (http://www.frac.info/), resistance risk of OXTP assumes to be medium to high (single site inhibitor) and therefore resistance management is required. There are several principal recommendations to delay the buildup of fungicide-resistant sub-populations in the field: avoiding the solo use of the fungicide at risk; minimizing the doses applied; avoiding curative applications; and, using mixtures or alternations with another fungicide having a different mode of action. Indeed, numerous studies showed the usefulness of dual or triple mixtures in suppressing the buildup of resistance in oomycete foliar pathogens against, e.g., phenyl amide fungicides in the field [see Gisi and Cohen [17]].

No studies are available on the efficacy of oxathiapiprolin-based fungicidal mixtures against foliar oomycete plant pathogens, including late blight in potato or tomato, either in growth chambers or in the field. The objective of the present study was to evaluate, in growth chambers and the field, the efficacy of OXPT and OXPT-based fungicides in controlling late blight induced in tomato by MFX-insensitive isolates of *P*. *infestans*. We show that mixtures provide enhanced control of the disease and therefore may delay the emergence and selection of OXPT-resistant subpopulations

## Materials and methods

### Growth chamber experiments

#### Fungicides

Syngenta Crop Protection, Stein, Switzerland, provided a gift of five solo fungicides, four oxathiapiprolin-based dual fungicidal mixtures and one blind formulation (480SL = BF) of mefenoxam. The five solo fungicides were oxathiapiprolin (OXPT) 250OD, chlorothalonil (CHT) 500SC, azozystrobin (AZ) 250SC, mandipropamid (MPD) 250SC, and mefenoxam (MFX) 480SL. The four dual pre-packed mixtures were OXPT+CHT 406SC, OXPT+AZ 170SC, OXPT+MPD 280SC, and OXPT+MFX 280DC. Table 1 presents the composition of the mixtures and the weight ratio between oxathiapiprolin (OXPT) and its partner fungicide in each mixture. The fungicides were suspended in water and diluted to a series of x10 fold concentration suspensions from 0.00001 to 1000 ppm. All doses are ppm ai (active ingredient, w/w). For the dual mixtures, an indicated concentration represents the combined concentrations of both ingredients.

**Table 1:** Composition of four ready-to-use dual mixtures of oxathiapiprolin.

Mixture	Components	Ratio,w/w	gai /kg^a^	Formulation
**OXPT+CHT**	oxathiapiprolin+chlorothalonil	1+66.7	6+400	406 SC
**OXPT+AZ**	oxathiapiprolin+azoxystronin	1+10.3	15+155	170 SC
**OXPT+MPD**	oxathiapiprolin+mandipropamid	1+8.33	30+250	280 SC
**OXPT+MFX**	oxathiapiprolin+mefenoxam	1+3	70+210	280 DC

^a^ gram active ingredient per kg product.

#### Plants

Tomato cv. Roter Gnom (deterministic growth type, gift from Syngenta Crop Protection, Stein, Switzerland) was used in all experiments. Potted plants were grown from seeds in a greenhouse in 250 ml pots filled with peat: vermiculite (3: 1, v/v) mixture, one plant per pot, and used at the 10-leaf stage, unless otherwise stated.

#### Sensitivity of *P*.*infestans* to oxathiapiprolin

We evaluated the sensitivity to OXPT of 106 Israeli isolates of *P*. *infestans*. Twenty, 49 and 37 isolates were collected from potato fields in the Western Vegev during 2016, 2017 and 2018, respectively. All were highly infectious to tomato, insensitive to mefenoxam and belonged to genotype 23A1 or 13A2. Sensitivity tests were done with detached tomato leaflets taken from adult tomato plants (leaves three and four from the top) growing in soil in a greenhouse. Leaflets were placed in Petri dishes on wet filter paper lower surface uppermost. Two assays were performed: preventive and curative. In the first, leaflets were sprayed with OXPT and inoculated at about 1h after spray. In the second, leaflets were sprayed with OXPT at 1-day post inoculation (1 dpi). Leaflets were sprayed with OXPT suspension (0.0001–10 ppm ai, 10-fold diluted concentrations) with the aid of a fine glass atomizer. Each leaflet was inoculated with six droplets (10 μl) containing 100 sporangia each, of an isolate of *P*. *infestans*, two leaflets/dish/ isolate/dose. Plates were incubated at 18°C in the dark for 15 h and then at 20°C, 12 h photoperiod, 60 μmoles.s^-1^.m^-2^. Percent of leaflet area showing sporulation of *P*. *infestans* was visually estimated at x10 magnification at 7 dpi (days post-inoculation). Minimal inhibitory concentration (MIC) was determined as the lowest dose at which no sporulation was visible.

#### Microscopy

Detached tomato leaflets (n = 4) were placed on wet filter paper in Nunk plates (20x20x2cm, Nunk, Denmark), drop-inoculated with sporangia of isolate 164 of *P*. *infestans* (insensitive to MFX, 23A1, collected in March 2016 from potato at Nirim, Western Negev), and sprayed with OXPT of various concentrations at 1 dpi. Leaf discs (12 mm diam.) were cut out at 2 dpi for microscopic observations. Diameter of lesions were measured and sporangial yield was determined by hemocytometer at 4 dpi. Leaf discs were clarified in boiling ethanol, placed on glass slides, lower surface uppermost, treated with 0.01% clacofluor (Sigma), and examined with an epi-fluorescent microscope (Olympus A70), as described before [18].

#### Translaminar efficacy of fungicides

Tomato leaflets (n = 4) were placed on a wet filter paper in each of two 14 cm Petri dishes, upper surface uppermost, and sprayed with a fungicide. After 2 h, leaflets in one plate were turned to face their lower surface uppermost. All leaflets were spray-inoculated with *P*. *infestans* (5x10^3^ sporangia per ml) at 3 h after fungicide application. Translaminar efficacy was determined at 7 dpi by estimating the proportion of leaf area showing sporulation of *P*. *infestans*.

#### Lateral efficacy of fungicides

Detached leaflets were placed on a dry paper towel, upper (n = 4) or lower (n = 4) surface uppermost. The left half of each leaf blade was covered with aluminum foil along the main vein, and the exposed half of the blade was sprayed with a fungicide. The aluminum foil removed and the leaflets placed on a wet filter paper in Nunk plates. Leaflets were then spray-inoculated with *P*. *infestans*. Lateral movement of the fungicides was determined at 7 dpi according to the area showing sporulation of *P*. *infestans* in each half of the leaflet. Similarly, in order to examine the proximal or distal movement of a fungicide in detached leaflets, the proximal half of the leaf blade adjacent to the petiole or its distal half was covered with aluminum foil before fungicide application.

#### Preventive efficacy in intact plants

Potted plants (n = 3–4) were sprayed with a fungicide suspension on their upper leaf surfaces with the aid of a fine glass atomizer. Control plants were left untreated. Control and treated plants were spray-inoculated two hours later with a sporangial suspension (5,000 sporangia/ml) of *P*. *infestans* (isolate 164) on their upper leaf surfaces, incubated in a dew chamber at 18°C in the dark for 15 h, and then transferred to a growth chamber at 20°C (60–70% RH, 12h light/day, 60 μEm^-2^s^-1^). Disease records were taken at 7 dpi (unless otherwise stated) by visual estimation of the percent of blighted leaf area per plant.

#### Curative efficacy in intact plants

Potted plants (n = 3–4) were inoculated in the manner described above. At one or two days after inoculation, they were sprayed with fungicides (as above) and returned to the growth chamber for symptom production. Disease records were taken at 7 dpi, as described above.

#### The effect of blind formulation on curative efficacy

Oxithiapiprolin was mixed with the soluble concentrate (SL) blind formulation of MFX (= BF) at a weight ratio of 1+3, OXPT+BF. Plants were inoculated with *P*. *infestans* and then sprayed curatively at one or 2 dpi with various doses of OXPT+BF, OXPT, or BF. Disease records (percentage blighted leaf area) were taken at 7 dpi as above.

#### Protection of newly developed leaves

Persistence of the fungicides on treated leaves and their systemic movement to newly developed leaves was tested as follows: 5-leaf tomato plants were sprayed with fungicides of 100-ppm ai and allowed to grow in a growth chamber at 20°C. At 17 days, when plants reached the 10-leaf stage, they were inoculated with *P*. *infestans*; percent blighted leaf area was recorded at 7 dpi in bottom and top leaves. In other experiments, 10-leaf plants were sprayed with 10-ppm ai of the fungicides and 8 days later, when five new leaves were developed, they were inoculated with *P*. *infestans*. Untreated plants served as controls. Disease records were taken from the treated and the untreated leaves at 7 dpi.

#### Efficacy in detached fruits

Green tomato fruits (about 5-cm diameter, n = 10) were sprayed with fungicides of 0.1, 1, or 10 ppm ai (except OXPT: 0.0001–10 ppm ai) and two hours later, inoculated with sporangia of *P*. *infestans* (preventive application). Another set of fruits was first inoculated, and at 2 dpi sprayed with fungicides at 1, 10, and 100 ppm ai (except OXPT: 0.01–100 ppm ai). Fruits were incubated at 20°C in the dark. The number of fruits showing late blight symptoms was determined at 8 dpi.

#### Efficacy of root treatment

Tests were performed with 5-leaf tomato plants. One ml fungicide suspension of 1, 10, or 100-ppm ai was pipetted onto the soil surface around the stem base of each plant (0.001, 0.01, and 0.1 mg ai/plant, 5 plants per dose). Five ml of water were applied to the soil around the stem base at 1 h and again at 5 h after fungicide application. The plants were inoculated with *P*. *infestans* one day after treatment, and percent of leaf area blighted in each plant was recorded after a week.

#### Efficacy of seed treatment

One hundred tomato seeds were mixed with 100 mg fungicide preparation (see Table 1) in a round-bottom glass flask and rotated at 500 rpm for one hour. Seeds were left on the bench for a week and then planted in pots containing peat mixture, 1 seed per pot. Untreated seeds served as controls. The emerging plants were grown in a growth chamber at 20°C and inoculated with *P*. *infestans* when they reached the fourth or sixth leaf stage. Percent of leaf area blighted was recorded for each plant at 7 dpi.

### Field experiments

Four field experiments were conducted during 2016–2018. All experiments were done at Bar-Ilan University Farm **(**32° 04' 2.40" N, 34° 50' 19.79" E) in 50x6 m net houses covered with white, 50 mesh, insect-proof plastic screens. Tomato plants (cv. Roter Gnom) were grown from seeds in Speedling trays, cell size 5x5 cm (Hishtil Ltd, Petach-Tikva, Israel) and planted in the field at the 10–12 leaf stage in 1 m^2^ plots, five plants per plot. Plants were drip-fertigated (0.05% 20:20:20 NPK solution) 2–3 times a day, depending on the season. Fungicides were applied to the plants at about 3–4 weeks after planting. Plants were sprayed with fungicides on their upper leaf surface with a hand sprayer at a rate of 50 ml per plot 1 m^2^. Untreated plots served as controls. Control and treated plants were artificially inoculated on the same day or one day after fungicide application by spraying sporangial suspension (1x10^4^ sporangia/ml) of *P*. *infestans* at a rate of 10 ml/1m^2^ plot. Inoculation was done at night with a single isolate or a mixture of several isolates (see below). All isolates used in the field were insensitive to MFX. To assure infection, plants were covered after inoculation with a plastic sheet until the next morning. Late blight records were taken at various time intervals after inoculation by visual assessment of the percent of blighted leaf area per plant. Percent of season-long protection for each fungicide was calculated as the ratio between the mean area under disease progress curves (AUDPCs) of all four doses of a fungicide and the AUDPC of the control fungicide-free plots. We designed the following formula to calculate it: Percent of season-long protection = 1-(mean AUDPC-treated /AUDPC control) x100.

### Statistics

Growth chamber experiments were repeated once or more. t-test analysis was performed to determine significant differences between means at α = 0.05. ED 50 and ED 90 values were derived from log-probit regression curves using SPSS software. Synergy was calculated using the Abbott or Wadley formulae as we described before [19, 20]. For all field experiments, t-test analysis was employed to the final disease scores to determine significant differences (α = 0.05) between control and fungicide(s) treatments.

## Results

### Growth chamber experiments

#### Sensitivity to OXPT in detached tomato leaflets

**Fig 1:**
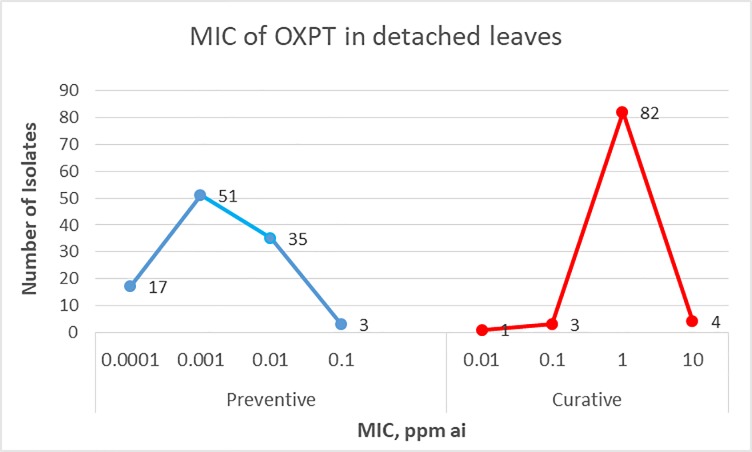
Minimal inhibitory concentration (MIC) values of oxathiapiprolin in preventive and curative application against late blight induced by Israeli isolates of *Phytophthora infestans*, in detached tomato leaves. A- Detached leaflets were treated with oxathiapiprolin of 0.00001–10 ppm active ingredient (ai) on lower leaf surface and thereafter spray-inoculated with each of 106 isolates of *P*. *infestans*. B- Detached leaflets were first inoculated with each of 90 isolates of *P*. *infestans* on lower leaf surface and at 1 day after inoculation were treated with oxathiapiprolin of 0.00001–10 ppm ai. Percent of leaf area showing sporulation of the pathogen was recorded at 7 days post-inoculation (dpi).

Fig 1 shows the preventive and curative (1 dpi) efficacy of OXPT against late blight induced by 106 and 90 field isolates of *P*. *infestans*, respectively. Minimal inhibitory concentration (MIC) values in preventive application ranged between 0.0001 and 0. 1-ppm ai: 17, 51, 35 and 3 isolates were fully inhibited at 0.0001, 0.001, 0.01 and 0.1 ppm ai, respectively. MIC values were x100-1000 higher in curative application, ranging between 0.01 and 10 ppm ai (91.1% of the isolates were fully inhibited at 1 ppm ai).

#### Microscopy

**Fig 2:**
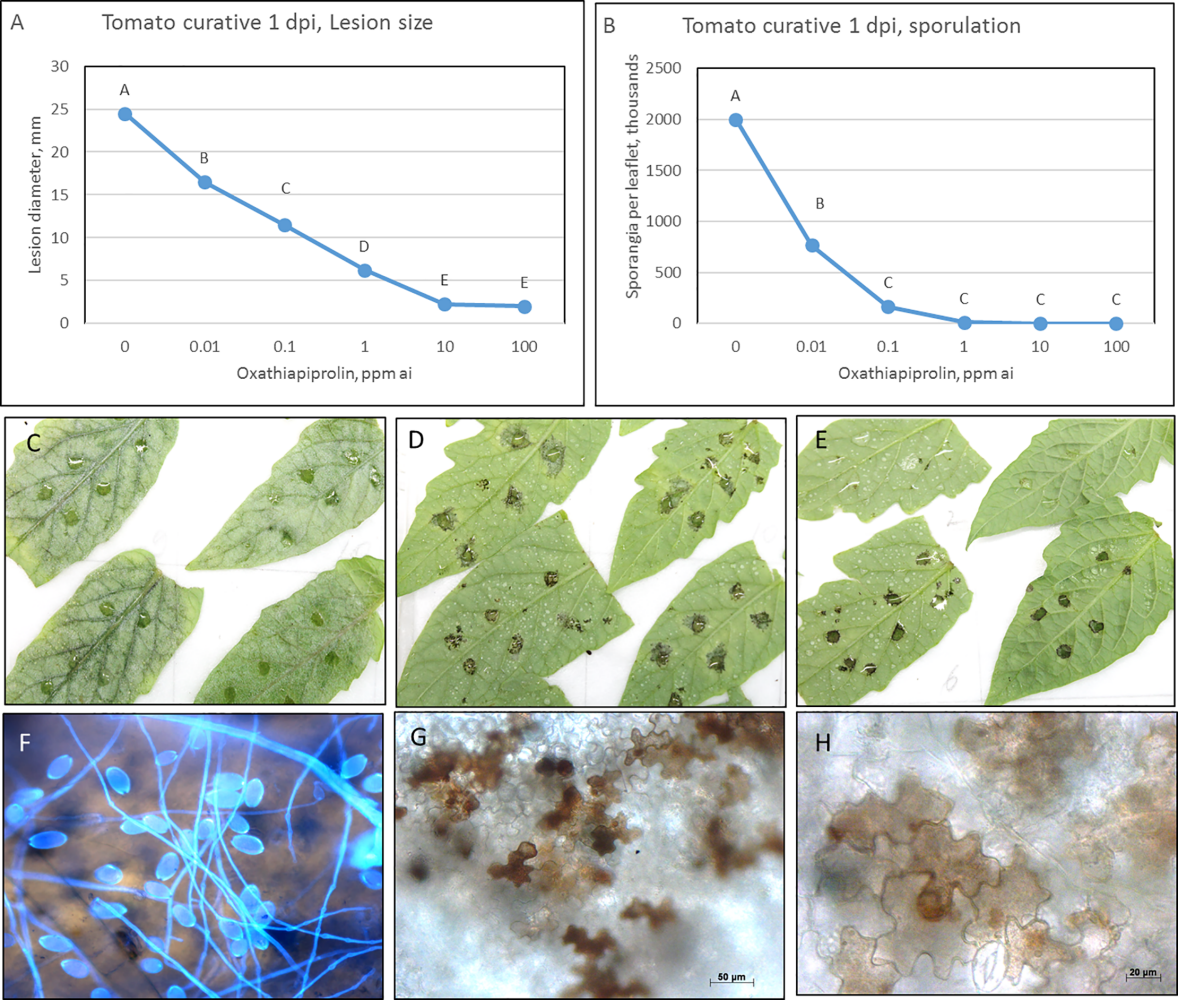
Efficacy (A-E) and microscopy (F-H) of curative application (1 dpi) of oxathiapiprolin on late blight development in detached tomato leaves. Detached tomato leaflets were drop-inoculated on their lower leaf surface with sporangia of *P*. *infestans* and sprayed with oxathiapiprolin at 1 dpi. Leaf discs were removed at 2 dpi for microscopic observations. Lesion size and sporangial yield were determined at 4 dpi. A- Lesion size. B- Sporangial yield per leaflet. C, F- Sporulation of *P*. *infestans* in control leaflets. D, G- Response of leaflets treated with 1-ppm ai oxathiapiprolin. E, H- Response of leaflets treated with 10-ppm ai oxathiapiprolin. Note the necrosis induced by oxathiapiprolin.

Curative application of OXPT to detached tomato leaves was associated with necrosis, resembling a hypersensitive response (HR). Curatively applied OXPT significantly reduced both lesion size and sporangial yield in a dose-dependent manner (Fig 2A and 2B). While control leaves showed profuse sporulation (Fig 2B, 2C and 2F), OXPT of ≥1 ppm ai induced strong necrosis and full suppression of sporulation (Fig 2B, 2D and 2E). The necrosis was restricted to the drop-inoculated sites, although OXPT was present all over the leaflet. Microscopic observations revealed necrotic epidermal and mesophyll cells only beneath the inoculated sites (Fig 2D, 2E, 2G and 2H), suggesting that OXPT induced necrosis in only the host cells that were penetrated by the pathogen.

#### Preventive efficacy of OXPT in intact plants

**Fig 3:**
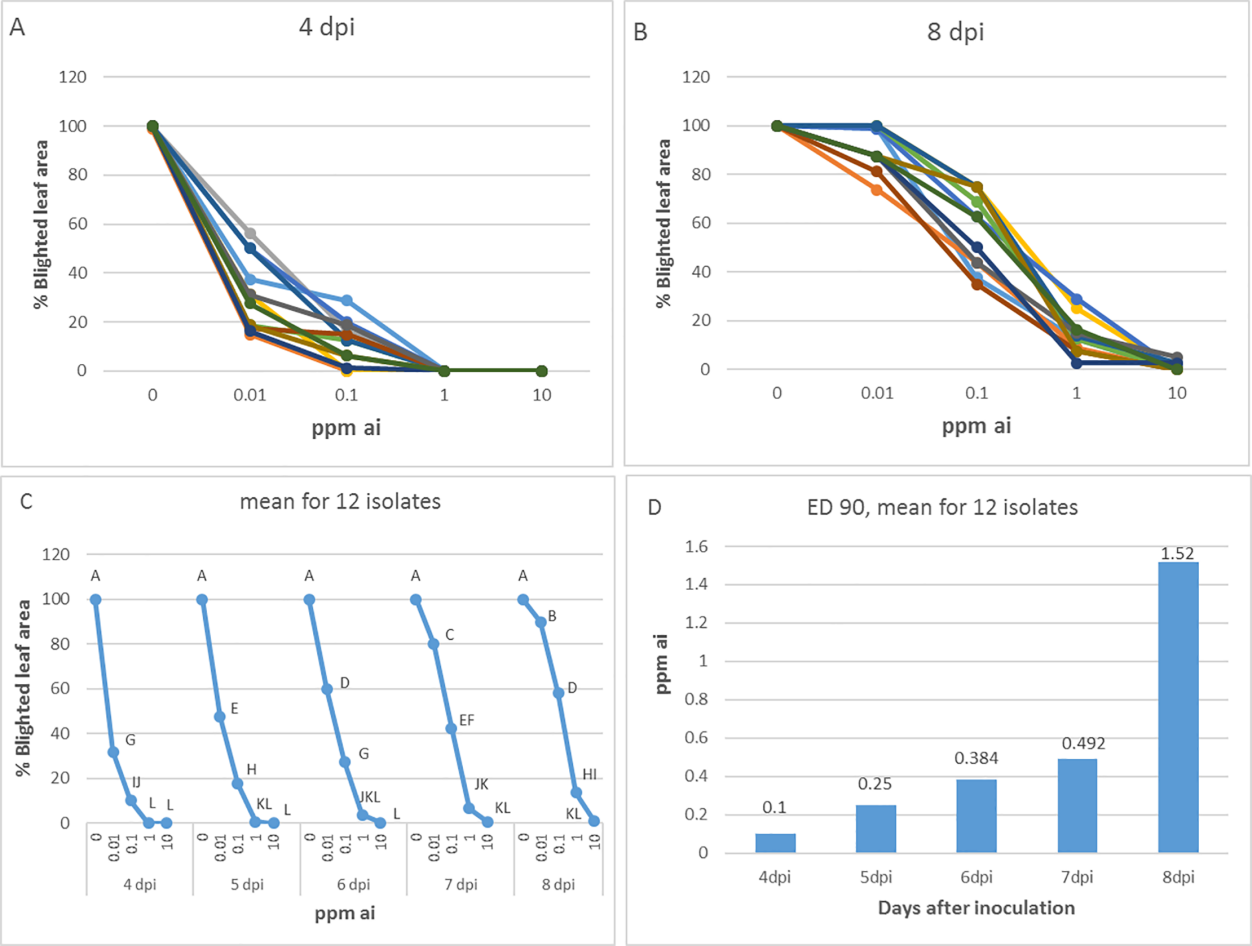
Preventive activity of oxathiapiprolin against late blight induced by 12 Israeli isolates of *P*. *infestans* in intact tomato plants. A- Percent of leaf area blighted for each isolate at 4 dpi. Note that minimal inhibitory concentration (MIC) = 1 ppm ai. B- Percent of leaf area blighted for each isolate at 8 dpi. Note that MIC≥10-ppm ai. C- Mean dose-response curves for 12 isolates at various time intervals after inoculation. D- ED 90 values derived from the values in presented C.

The preventive efficacy of OXPT against late blight induced by each of 12 isolates (4 isolates per year) of *P*. *infestans* in 10-leaf potted tomato plants is shown in Fig 3. Percent of blighted leaf area was recorded daily from 4 dpi to 8 dpi. The results show that the efficacy of OXPT depends on the isolate and on the duration of the colonization period (Fig 3A and 3B). Mean MIC values at 4, 5, 6, 7, and 8 dpi were 1, 1, 10, >10 and >10 ppm ai, respectively (Fig 3C). Fig 3D shows that the mean ED 90 increased as duration of the colonization period prolonged.

#### Preventive and curative efficacy of OXPT and oxathiapiprolin mixtures in intact plants

**Fig 4:**
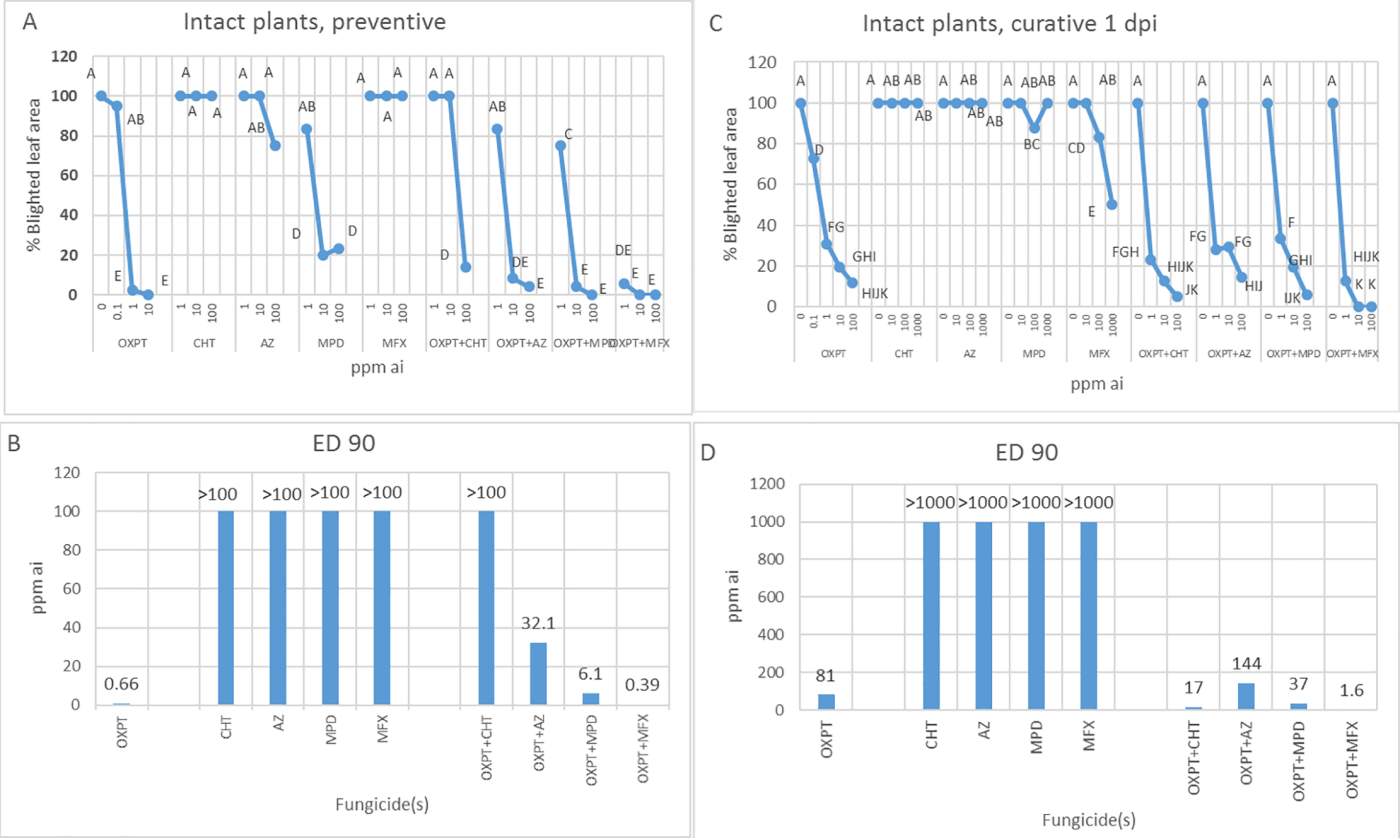
Preventive (A and B) and 1 dpi curative (C and D) activities of oxathiapiprolin (OXPT), chlorothalonil (CHT), azoxistrobin (AZ), mandipropamid (MPD), mefenoxam (MFX), and their dual mixtures OXPT+CHT, OXPT+AZ, OXPT+MPD, and OXPT+MFX against late blight induced by isolate 164 of *P*. *infestans* in intact tomato plants. A, C- Percent of leaf area blighted at 10 dpi. B, D- Effective dose 90% values (ED 90) calculated from the 10 dpi data. Different letters in A and C indicate significant differences between means (t-test, α = 0.05). Note the outperformance of OXPT+MFX.

The preventive efficacy of OXPT, CHT, AZ, MPD, MFX, and their dual mixtures OXPT+CHT, OXPT+AZ, OXPT+MPD, and OXPT+MFX against the MFX-resistant, highly aggressive isolate 164 of *P*. *infestans* in intact tomato plants is shown in Fig 4A. The ED 90 values, derived from the data in Fig 4A, are shown in Fig 4B. They were used to calculate the synergy factor (SF, Wadley formula) between OXPT and its partners. The results suggested a synergistic interaction between OXPT and MFX, with a SF value of 6.6. The 1 dpi curative efficacy of the same fungicides are shown in Fig 4C and 4D. Higher doses of OXPT or mixtures were required to control the disease with curative application when the pathogen was already developing inside the plant. OXPT provided 88% control of the disease at 100-ppm ai whereas the other solo fungicides showed no efficacy at this dose. The mixture OXPT+MFX was superior to OXPT, providing 100% control at 10-ppm ai as against the other mixtures that provided 85–95% control at 100-ppm ai (Fig 4C). ED 90 value for OXPT+MFX was smallest (Fig 4D).

In other experiments, the fungicides were applied 2 days after inoculation at a dose of 1, 10, and 100-ppm ai. Results (not shown) indicated ~30% control of the blight by OXPT of 100-ppm ai. The mixture OXPT+MFX outperformed all fungicides, providing 77 and 97% control of the disease at 10 and 100-ppm ai, respectively. All other fungicides provided 0–15% control of the disease.

**Fig 5:**
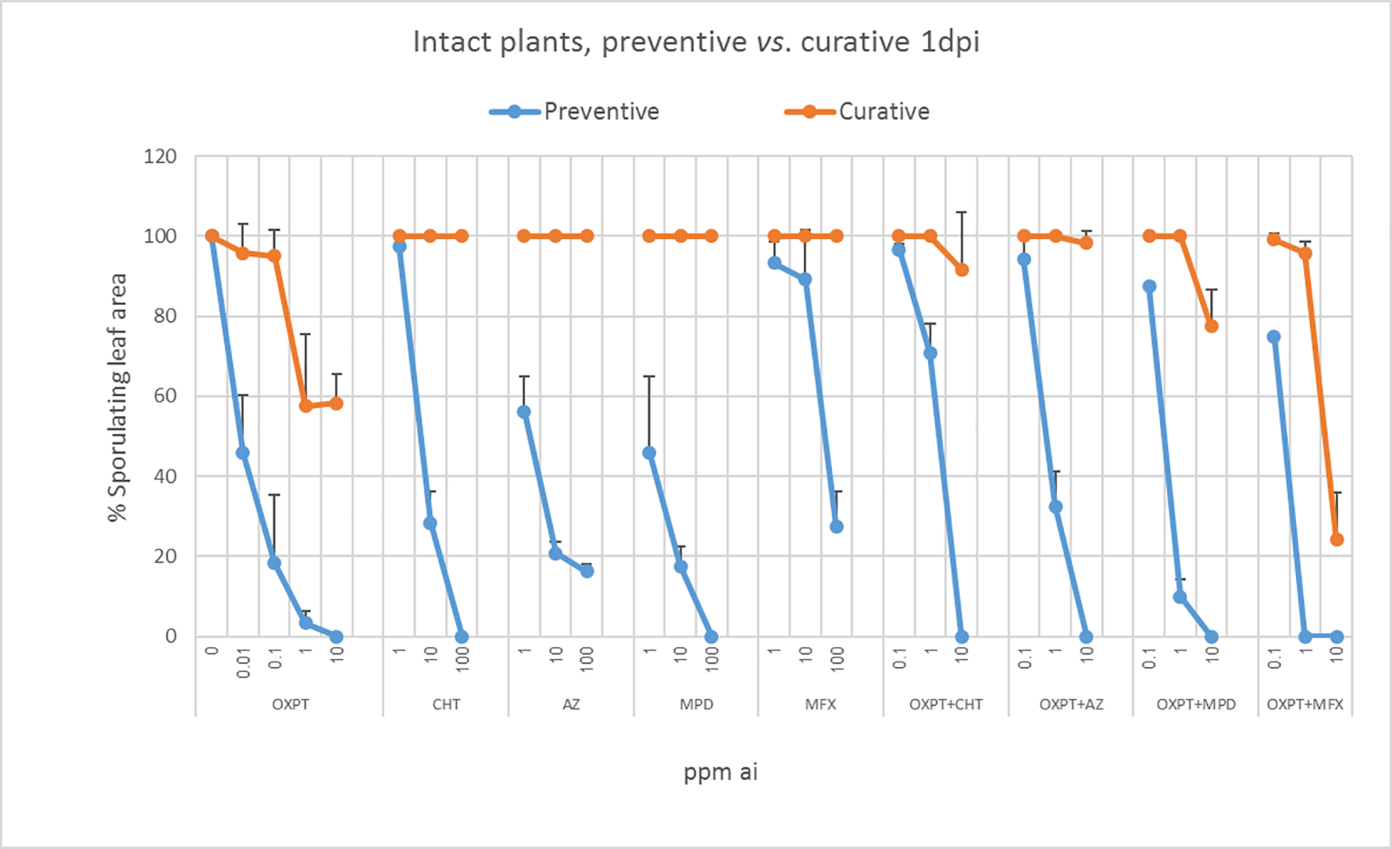
Comparison between preventive (blue lines) and curative (red lines) activity of OXPT, CHT, AZ. MPD, MFX and their dual mixtures OXPT+CHT, OXPT+AZ, OXPT+MPD and OXPT+MFX in controlling late blight induced by isolate 164 of *P*. *infestans* in intact tomato plants. Note the reduced efficacy of all fungicides in curative application and the outperformance of OXPT+MFX.

We compared the preventive and curative efficacies of all nine fungicides in one experiment. Curative (1 dpi) application was much less effective compared to preventive application for all fungicides. OXPT+MFX outperformed in both assays (Fig 5).

#### Translaminar activity in detached leaves

**Fig 6:**
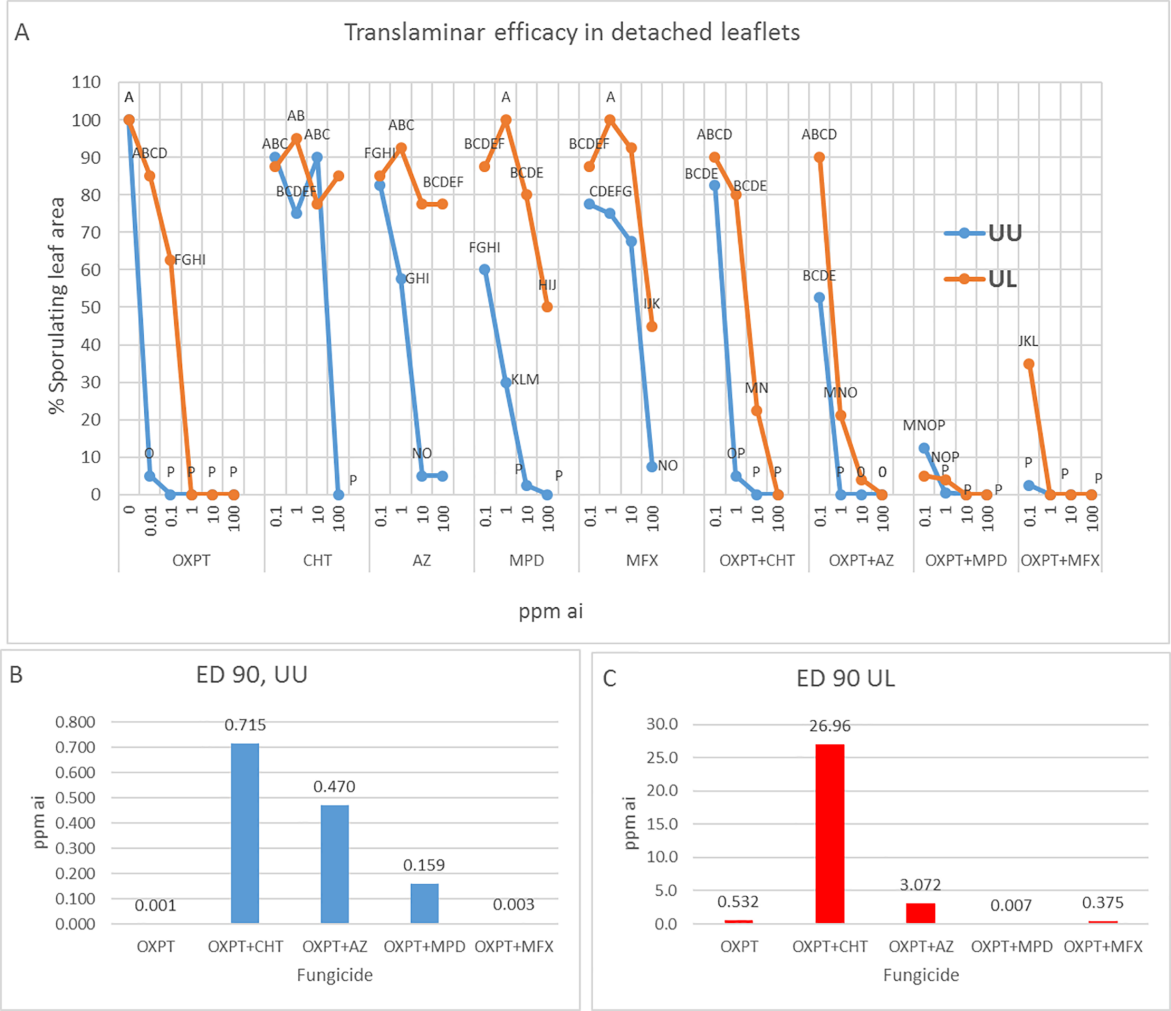
Translaminar activity of OXPT, CHT, AZ. MPD, MFX and their dual mixtures OXPT+CHT, OXPT+AZ, OXPT+MPD, and OXPT+MFX against late blight induced by isolate 164 of *P*. *infestans* in detached tomato leaves. A- Percent sporulating leaf area. UU = upper leaf surface treated upper leaf surface inoculated. UL = upper leaf surface treated lower leaf surface inoculated. Different letters indicate significant differences between means (t-test, α = 0.05). B and C- ED 90 values derived from A for UU or UL treatments.

Results given in Fig 6 show that fungicides were significantly more active in upper surface treated-upper surface inoculated (UU) treatments than in upper surface treated-lower surface inoculated (UL) treatments (Fig 6A). This indicates a partial translaminar movement of all fungicides across the leaf lamina. Oxathiapiprolin and OXPT+MFX provided the smallest ED 90 values in UU treatments (Fig 6B), whereas OXPT+MPD and OXPT+MFX provided the smallest ED 90 values in UL treatments (Fig 6C). OXPT+CHT was least effective in both UU and UL treatments (Fig 6B and 6C). The solo fungicides CHT, AZ, MDP, and MFX were moderately active in UU treatments but poorly active in UL treatments (Fig 6A).

#### Lateral efficacy of fungicides

Oxathiapiprolin and its mixtures exhibited poor lateral, proximal, or distal mobility in detached tomato leaflets. When applied at 100 ppm ai to one half of a detached leaflet (left, right, top, or bottom) they provided full control of the disease in the treated half leaflet, but poor or no control of the disease in the untreated half leaflet

#### Blind formulation influences efficacy

**Fig 7:**
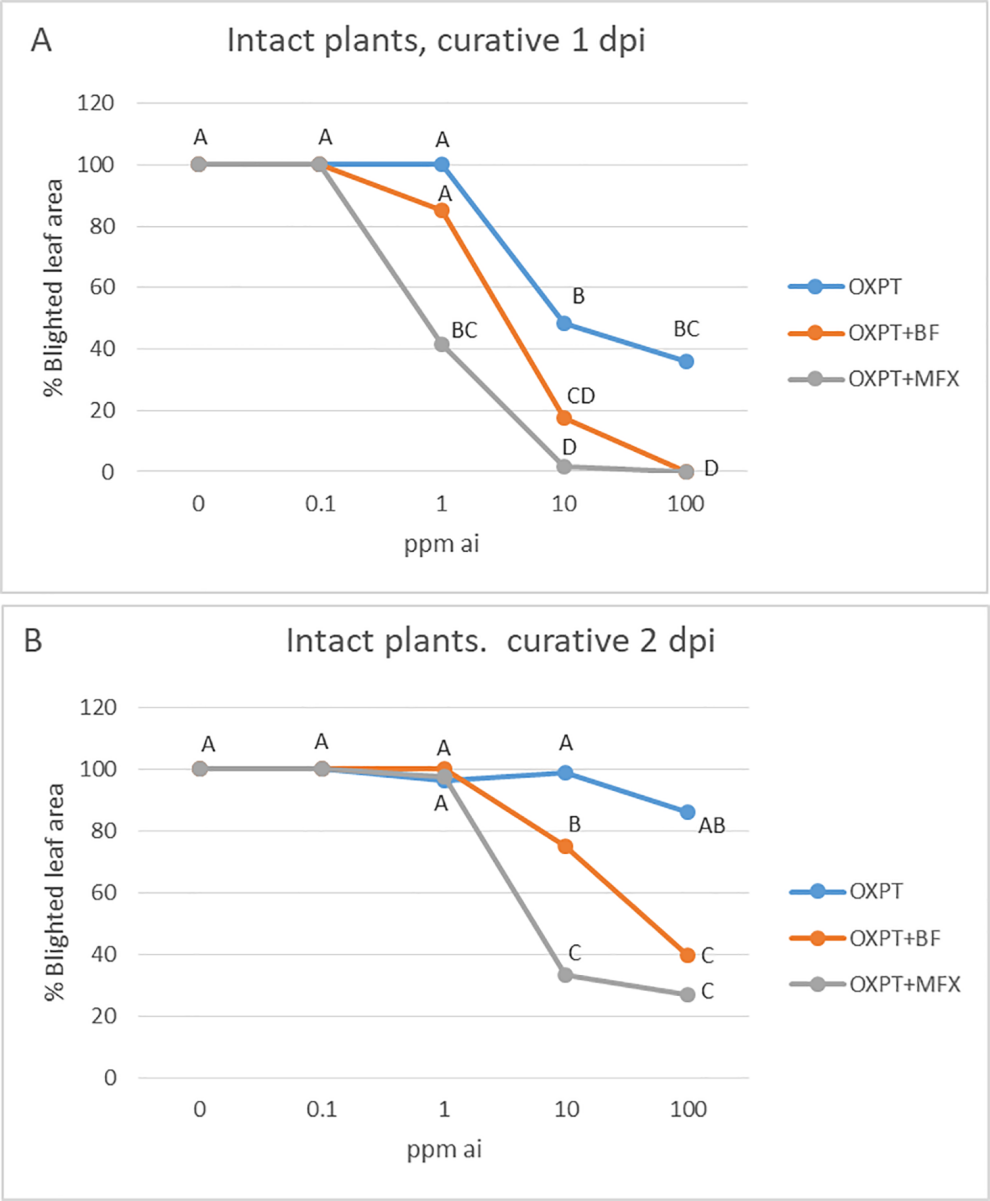
The enhancing effect of soluble concentrate (SL) blind formulation (BF) on the curative activity of OXPT against late blight induced by isolate 164 of *P*. *infestans* at 1 dpi (A) and 2 dpi (B). OXPT+BF = OXPT and BF mixed at a ratio of 1+3 by weight. Note the enhanced activity of OXPT+BF relative to OXPT, and of OXPT+MFX relative to OXPT+BF. Different letters indicate significant differences between means (t-test, α = 0.05).

The results presented in Fig 7 show that OXPT+BF is more effective than OXPT, and OXPT+MFX is more effective than OXPT+BF, suggesting that both, the soluble concentrate (SL) blind formulation and the intrinsic interaction between OXPT and MFX are responsible for the enhanced activity of OXPT+MFX.

#### Protection of new growth

**Fig 8:**
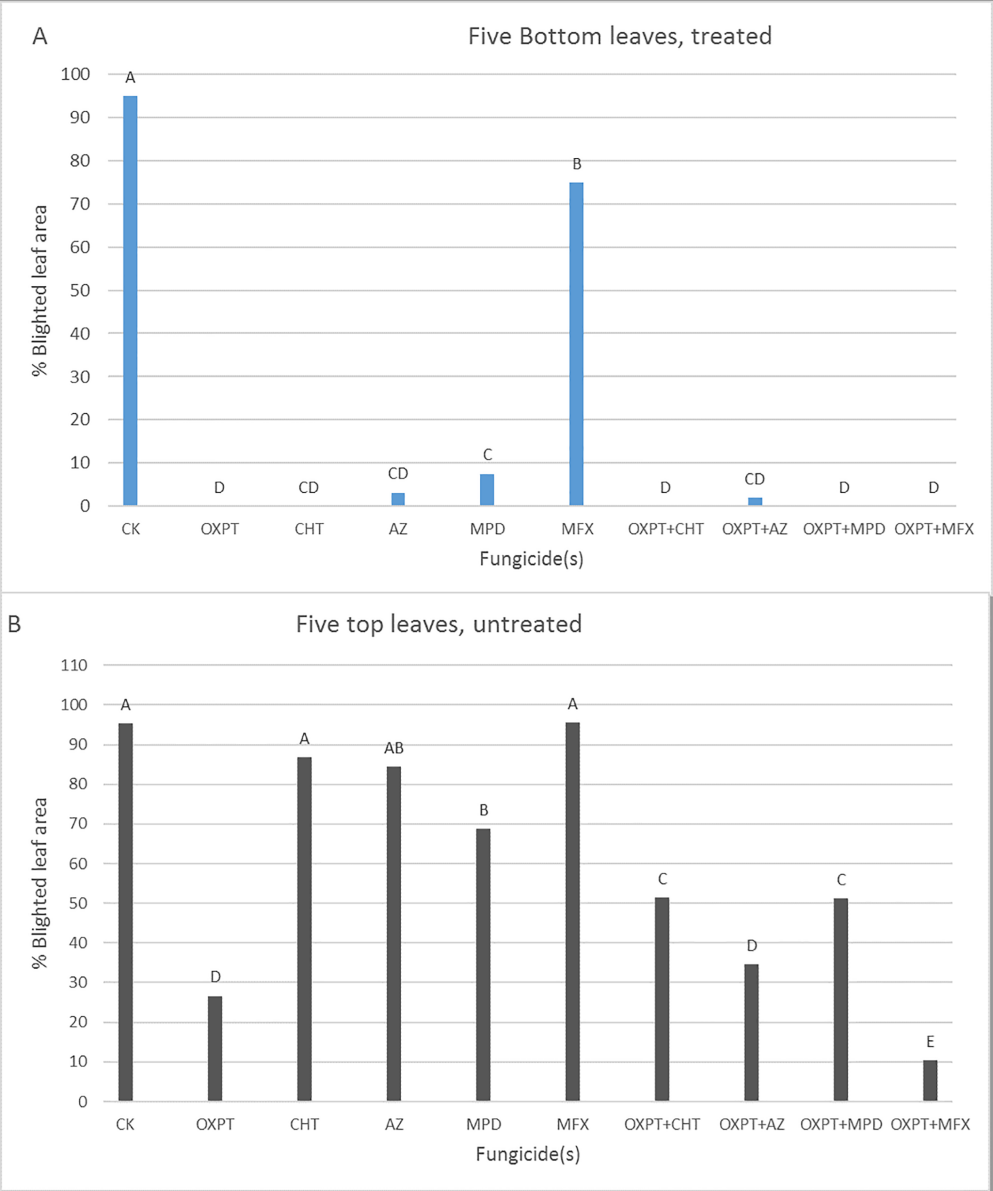
Systemic protection of the newly developed leaves of potted tomato plants from late blight. OXPT, CHT, AZ, MPD, MFX, OXPT+CHT, OXPT+AZ, OXPT+MPD, or OXPT+MFX were sprayed at 100-ppm ai onto 5-leaf plants. The plants were inoculated with isolate 164 of *P*. *infestans* 17 days later, at the 10-leaf stage. A- Percent of leaf area blighted at 7 dpi on the five bottom-treated leaves. B- Percent of leaf area blighted at 7 dpi on the top five newly grown leaves. Different letters above columns indicate significant differences between means (t-test, α = 0.05). Note that OXPT+MFX provided the best systemic protection.

Fungicides (100-ppm ai) persisted well on the five bottom-treated leaves of 10-leaf tomato plants, providing high protection against the blight at 10 days after application (Fig 8A). Solo MFX failed to do that due to the intrinsic insensitivity to MFX of the pathogen. However, fungicides were poorly effective in protecting the upper, five newly developed leaves. OXPT+MFX was significantly the most effective product in protecting the new growth from the blight (89.2%) (Fig 8B).

In other experiments, 10-leaf plants were treated with fungicides of 10-ppm ai. Percent of protection observed in the five newly-developed leaves was 82, 100, 64, 71, and 50% in plants treated with OXPT, OXPT+MFX, OXPT+CHT, OXPT+AZ, and OXPT+MPD, respectively. This indicated that OXPT+MFX was the only fungicide that could fully protect the new developing leaves, including the apical meristem of actively growing plants. The four partner fungicides provided no protection of the new growth.

#### Root treatment

**Fig 9:**
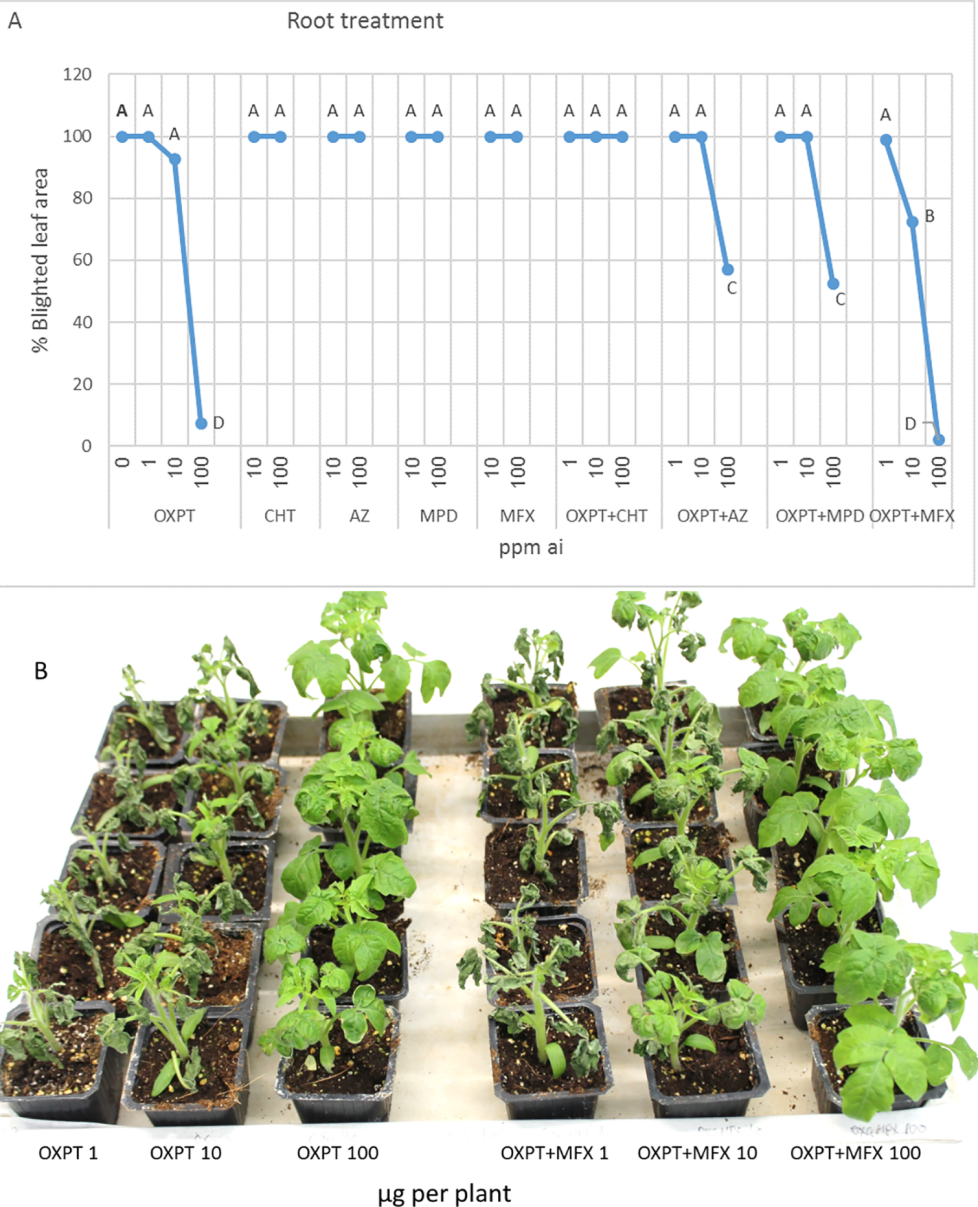
Systemic protection of tomato plants against late blight by root treatment with OXPT, CHT, AZ, MPD, MFX, OXPT+CHT, OXPT+AZ, OXPT+MPD, or OXPT+MFX. One ml of fungicide(s) suspension of various doses was applied to the soil around the stem base and one-day later plants were inoculated with isolate 164 of *P*. *infestans*. A- Percent of leaf area blighted taken at 7 dpi. Different letters indicate significant differences between means (t-test, α = 0.05). B- The appearance of inoculated plants at 7 dpi. Note the high protection provided by 100-ppm ai of OXPT or OXPT+MFX.

Five-leaf tomato plants were treated with 1 ml fungicide suspension around the stem base and inoculated with *P*. *infestans* one day later. The results in Fig 9 show that OXPT and OXPT+MFX were the only fungicides that were highly suppressive to the blight (Fig 9A and 9B).

#### Seed treatment

**Fig 10:**
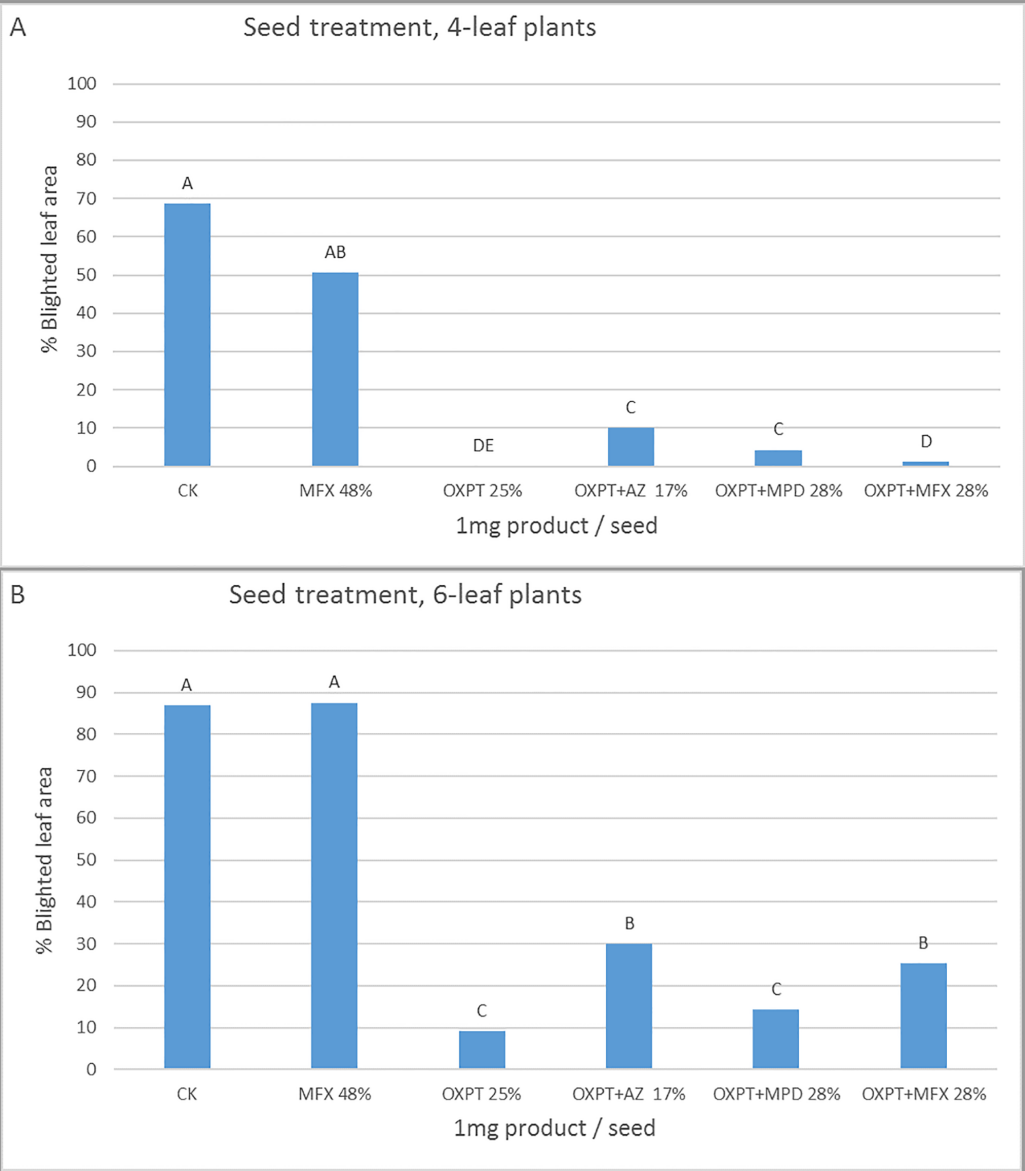
Systemic protection of tomato plants against late blight by seed treatment with OXPT, MFX, OXPT+AZ, OXPT+MPD, or OXPT+MFX. A- Plants grown from treated seeds were inoculated at the 4-leaf stage. B- Plants grown from treated seeds were inoculated at the 6-leaf stage. Different letters above columns indicate significant differences between means (t-test, α = 0.05).

Seeds treated with fungicide mixtures of 1 mg product/seed effectively protected the emerging tomato plants from late blight (Fig 10). The most effective products at the 4- and 6-leaf stage were OXPT and OXPT+MFX (Fig 10A), and OXPT and OXPT+MPD (Fig 10B), respectively. Plants inoculated at the 4-leaf stage were better protected (Fig 10A) compared to plants inoculated at the 6-leaf stage (Fig 10B), probably due to dilution effect.

#### Protection of tomato fruits

Higher doses of the fungicides were required to achieve complete control of late blight in tomato fruits as compared to tomato leaves. In preventive and curative spray applications, the most effective fungicide was OXPT+MFX, providing MIC of 1 and 100 ppm ai, respectively. Corresponding values for OXPT were 10 and >100 ppm ai, respectively.

### Field experiments

**Fig 11:**
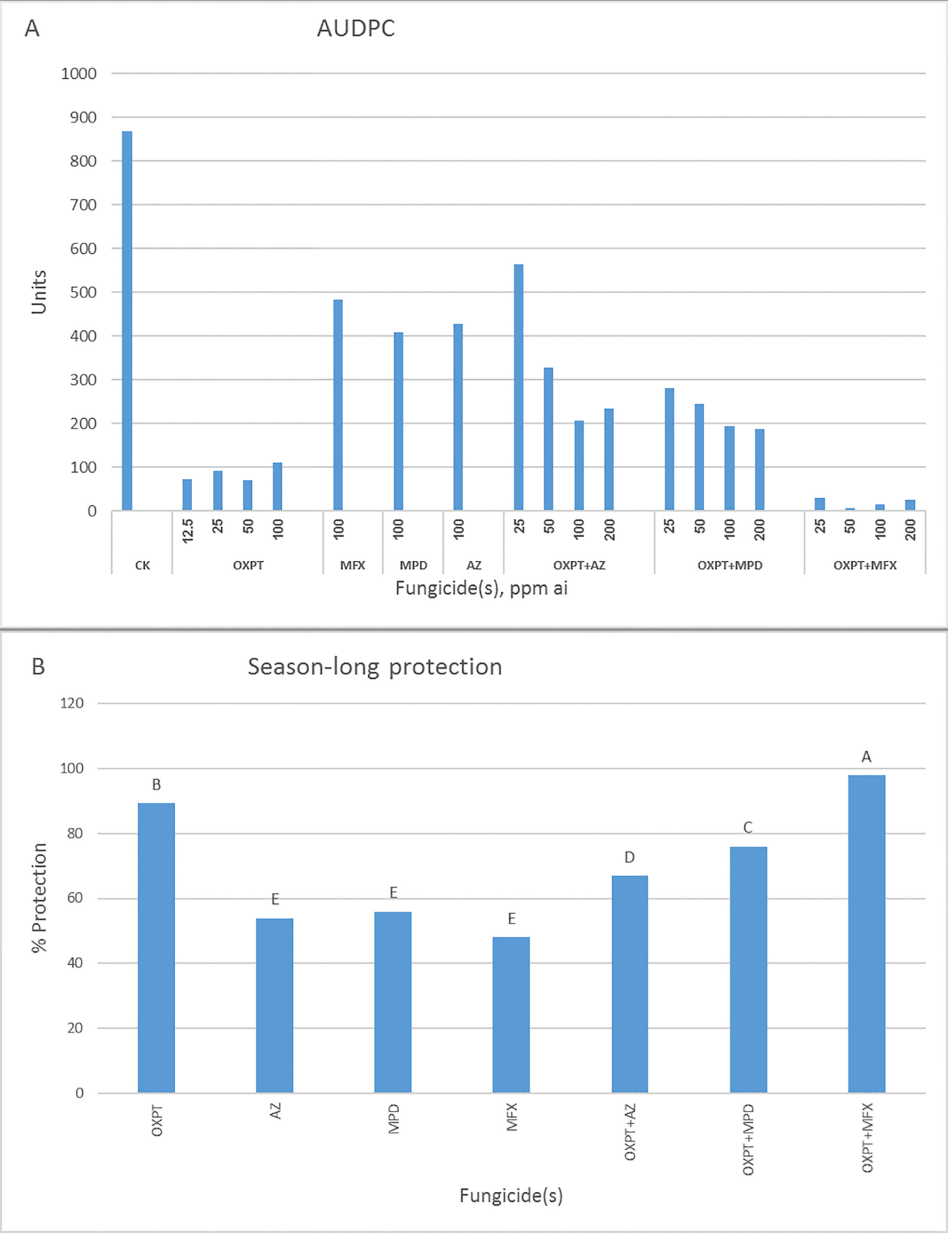
Control of late blight in tomato by OXPT, AZ, MPD, MFX and their dual mixtures under field conditions. Tomato plants were planted in the field on 25.9.2016, in two replicate plots of 1 m^2^, 6 plants per plot. One month later, plants were sprayed once with fungicides. One day later, plants were artificially inoculated with a mixture of 30 MFX-insensitive isolates of *P*. *infestans*. Percent leaf area blighted scores from each plant were taken at 4, 6, 7, 11, and 14 dpi. A- Area under disease progress curve (AUDPC) for each dose/fungicide. B- Percent of season-long protection provided by each fungicide. Different letters on columns indicate significant differences between treatments (t-test, α = 0.05). Note the outperformance of OXPT+MFX.

**Fig 12:**
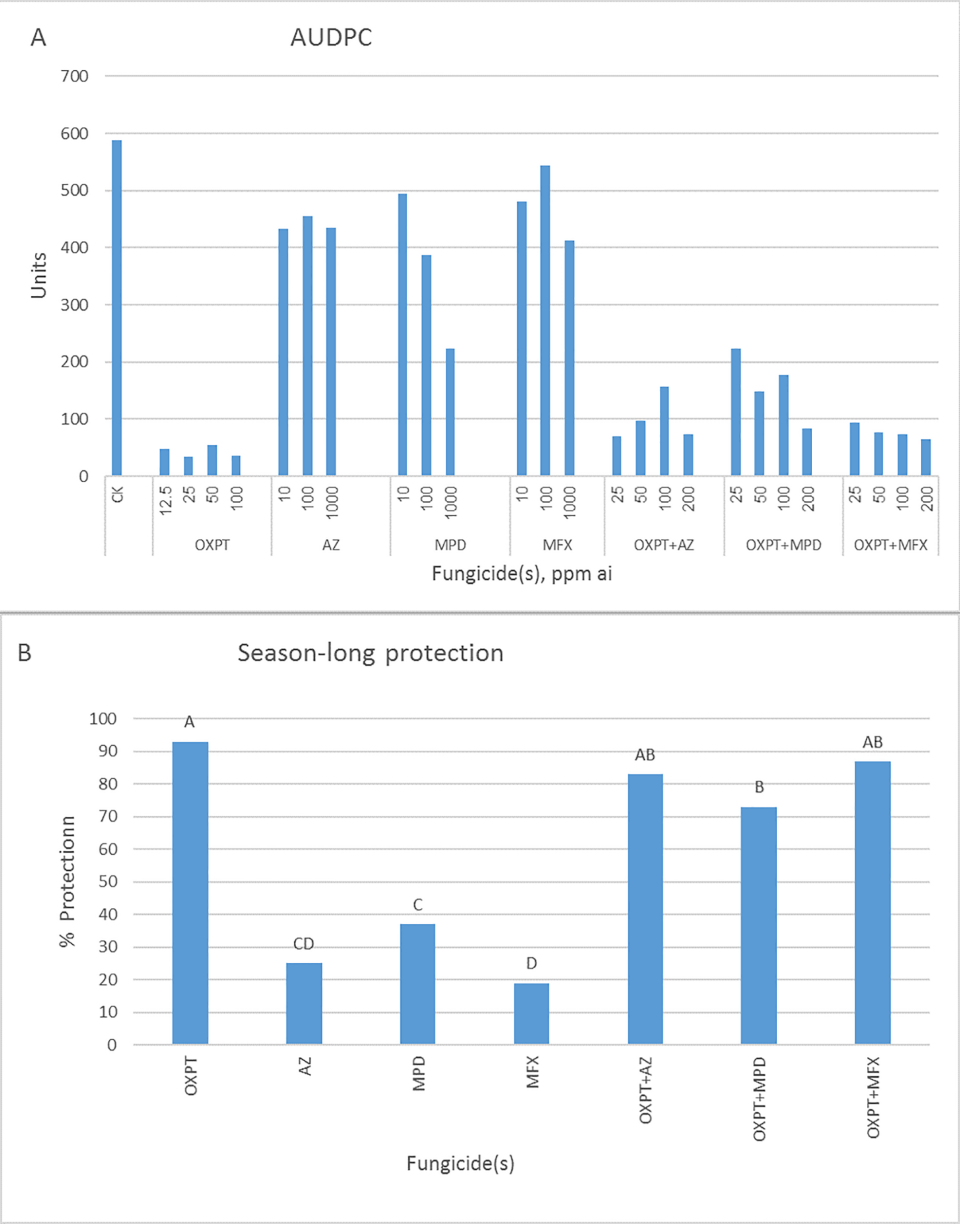
Control of late blight in tomato by OXPT, AZ, MPD, MFX, and their dual mixtures under field conditions. Tomato plants were planted in the field on 1.10.2016 in two replicated plots of 1 m^2^, 5 plants per plot. One month later, plants were sprayed once with fungicides. One day later, plants were artificially inoculated with the MFX-insensitive isolate 164 of *P*. *infestans*. Percent leaf area blighted scores were taken at 6, 10, and 13 dpi. A- AUDPC value for each dose/fungicide. B- Percent of season-long protection provided by each fungicide. Different letters on columns indicate significant differences between means (t-test, α = 0.05).

**Fig 13:**
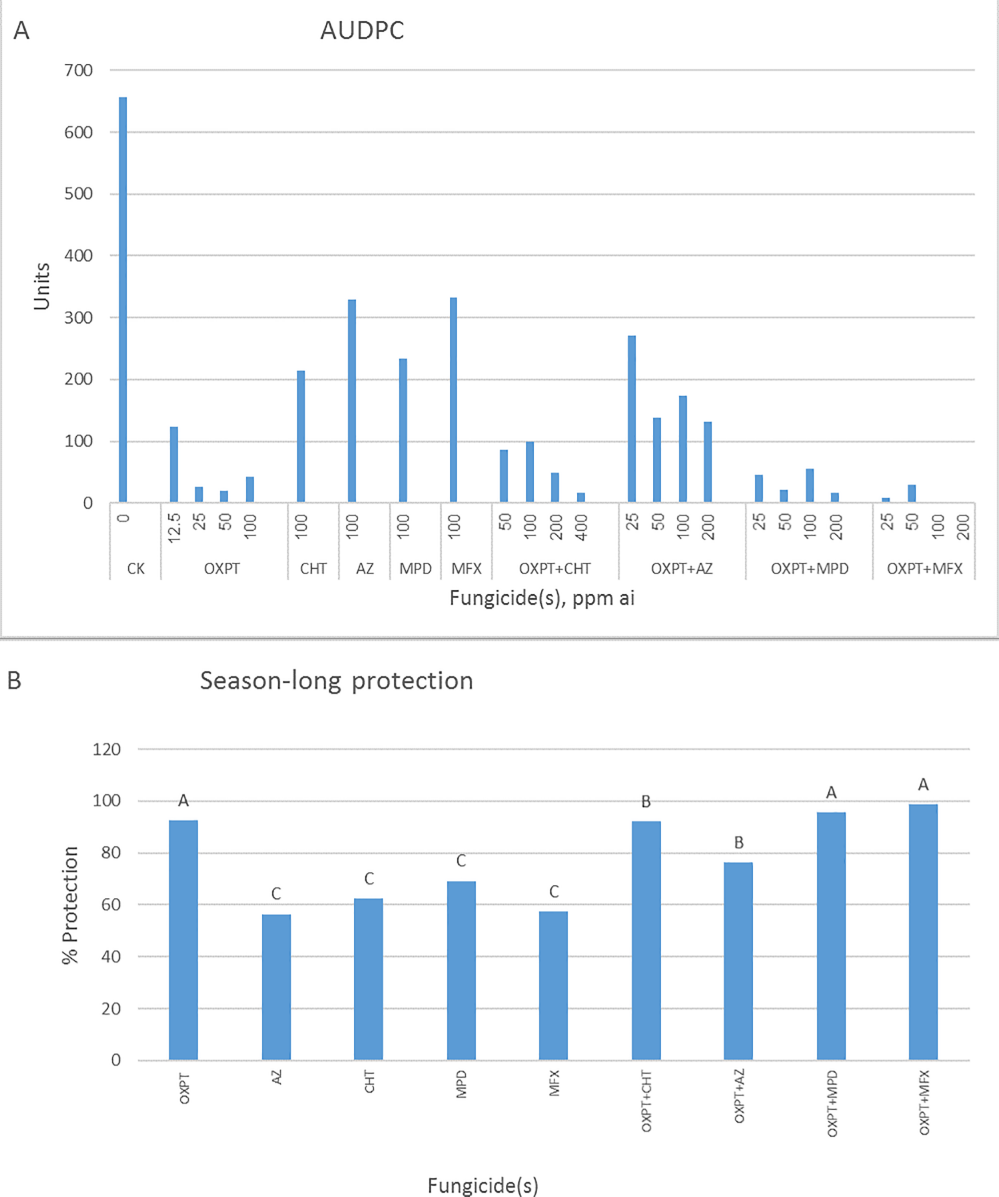
Control of late blight in tomato by OXPT, CHT, AZ, MPD, MFX, and their dual mixtures under field conditions. Tomato plants were planted in the field on 1.12.2017 in two replicate plots of 1 m^2^, 5 plants per plot. After 3 weeks, plants were sprayed once with fungicides. On the same day, the plants were artificially inoculated with a mixture of five MFX-insensitive isolates of *P*. *infestans*. Percent leaf area blighted scores were taken at 7, 10, 15, and 17 dpi. A- AUDPC values for each dose/fungicide. B- Percent of season-long protection provided by each fungicide. Different letters on columns indicate significant differences between means (t-test, α = 0.05).

**Fig 14:**
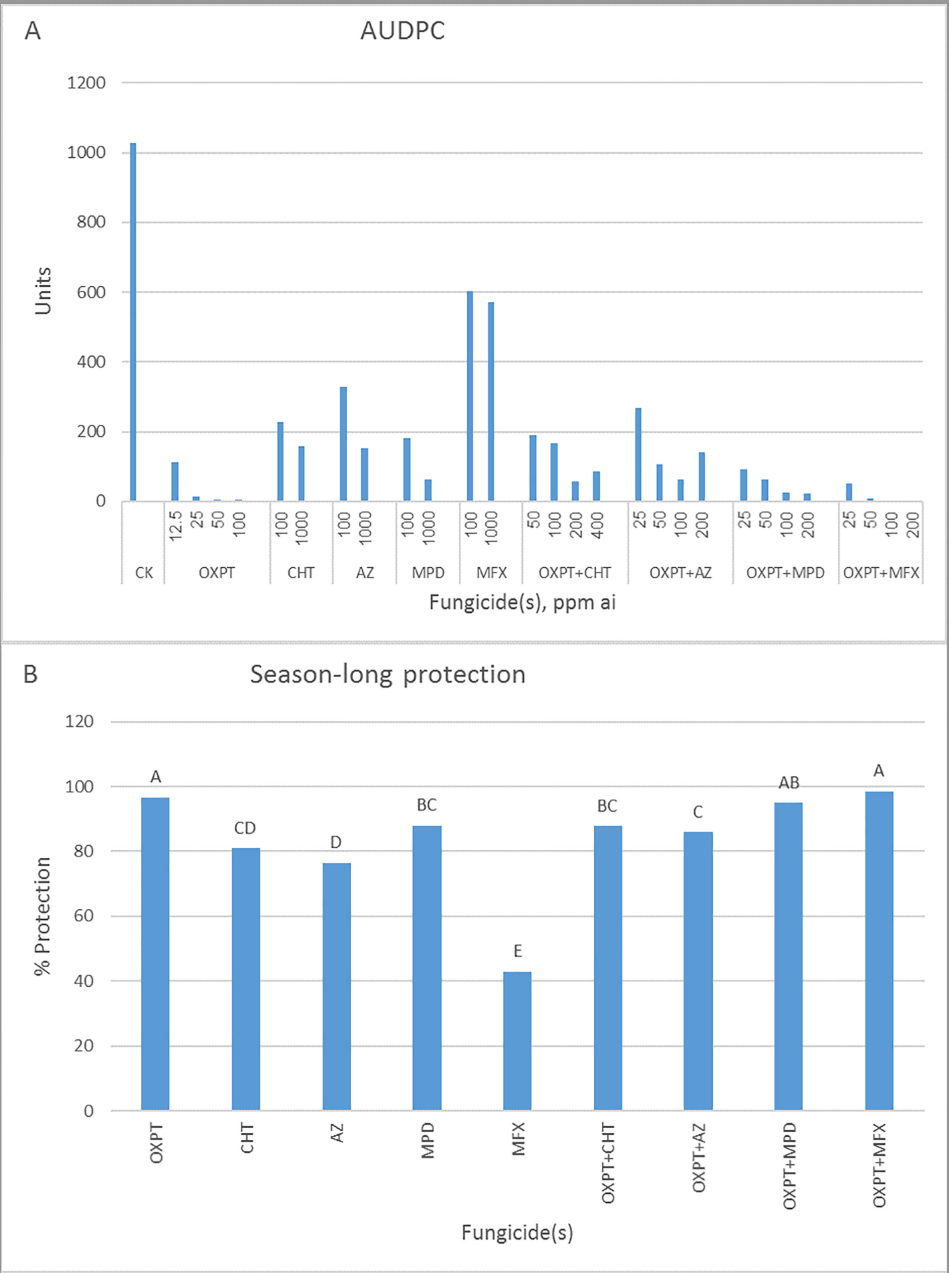
Control of late blight in tomato by OXPT, CHT, AZ, MPD, MFX, and their dual mixtures under field conditions. Tomato plants were planted in the field on 1.2.2018 in two replicate plots of 1 m^2^, 5 plants per plot. After 3 weeks, plants were sprayed once with fungicides. On the same day, the plants were artificially inoculated with a mixture of five MFX-insensitive isolates of *P*. *infestans*. Percent leaf area blighted scores were taken at 9, 12, 15, and 19 dpi. A- AUDPC values for each dose/fungicide. B- Percent of season-long protection provided by each fungicide. Different letters on columns indicate significant differences between means (t-test, α = 0.05).

Control of tomato late blight in four field experiments by OXPT and its mixtures are shown in Figs 11–14. For each experiment, AUDPC and percent of season-long protection for each fungicide(s) are given. In the first experiment, OXPT+MFX performed best, exceeding OXPT and the other two mixtures (Fig 11A and 11B). Thus, based on the AUDPC values, the control **e**fficacy of OXPT ranged between 86 and 91%, OXPT+MFX between 97 and 99%, OXPT+MPD between 74 and 80%, and OXPT+AZ between 54 and 77% (Fig 11A). The corresponding season-long protection values were 90, 98, 74, and 61%, respectively (Fig 11B). AZ, MPD, and MFX provided 51, 53, and 48% control, respectively. Control efficacy values obtained with 100-ppm ai were used to calculate the synergy factor (Abbott formula) between the fungicides in a mixture. Synergy factor values were 1.04, 0.87, and 0.69 for OXPT+MFX, OXPT+MPD, and OXPT+AZ, respectively, suggesting a synergy between OXPT and MFX.

In the second experiment, OXPT performed best (Fig 12A and 12B), providing season-long protection of 93%. The partner fungicides MFX, AZ, and MPD provided 19, 25, and 37% control, respectively and the mixtures OXPT+MPD, OXPT+AZ, and OXPT+MFX provided 73, 83, and 87% control, respectively (Fig 12B).

In the third field experiment, OXPT+MFX was the most effective fungicide, being the only product providing complete control of the blight at 100 ppm ai (Fig 13A). Season-long protection values for OXPT, OXPT+CHT, OXPT+AZ, OXPT+MPD, and OXPT+MFX were 93, 92, 77, 96, and 99%, respectively (Fig 13B).

In the fourth experiment, OXPT+MFX was the most effective fungicide, being the only product providing complete control of the blight at 100 ppm ai (Fig 14A). It also provided the highest (99%) season long protection (Fig 14B).

At the end of each field experiment, we collected infected leaves from the OXPT-treated plants, allowed them to sporulate and the sporangia of *P*. *infestans* were assayed for sensitivity to OXPT. All isolates (five per experiment) were sensitive to OXPT showing MIC of 0.001–0.01 ppm ai.

## Discussion

In our previous study [4], oxathiapiprolin was shown to effectively inhibit all developmental stages in the asexual life cycle of *Pseudoperonospora cubensis*, the downy mildew agent in cucurbit leaves. The fungicide was highly effective not only in preventive treatments but also in post-infection, curative applications. The post-infection activity was attributed to the sensitivity of all fungal structures to oxathiapiprolin.

In the present study, we confirm similar high efficacy of oxathiapiprolin [4] against *Phytophthora infestans*, the causal agent of late blight in tomato and potato. Sensitivity tests done *in vivo* with 106 local isolates of the pathogen showed MIC values of 0.0001–0.1 and 0.01–10 ppm ai in preventive and curative applications, respectively. The MIC dose in curative application was x100-1000 higher compared to preventive application, suggesting on partial uptake and/or reduced sensitivity to oxathiapiprolin of the mycelium relative to the sporangia.

A unique feature seen in detached tomato leaves when treated curatively with oxathiapiprolin was that leaves developed necrotic spots (similar to HR) only underneath the drop-inoculated sites. Oxathiapiprolin kills the initial haustoria of the pathogen inside the penetrated host cells and as a result, the cells die, producing necrosis. In cucumber and basil, minute, chlorotic, sterile lesions are produced as a consequence of curative application of oxathiapiprolin [4]. Interestingly, oxathiapiprolin is inhibitory to *P*. *infestans* in tomato even when applied at 2 days after inoculation, though higher doses are required.

We show here that besides its remarkable preventive and curative activities in tomato leaves, plants, and fruits, oxathiapiprolin has translaminar activity in detached leaves, movement from treated leaves to newly grown leaves, and systemic translocation from treated seeds or roots to leaves. Similar systemic activities of oxathiapiprolin were shown in cucumber against downy mildew [4], including translocation from the petiole to the leaf blade [10]. Surprisingly, we noticed very limited translocation of oxathiapiprolin in lateral, proximal, or distal directions in detached leaf blades of tomato. When each fungicide was applied at a high concentration of 100-ppm ai to one-half of the leaf blade (left, right, bottom, or top), the untreated half of the blade remained mostly susceptible to the disease.

Oxathiapiprolin is a site-specific fungicide. The appearance of resistant mutants, therefore, is expected. So far, no resistance against oxathiapiprolin was reported in *P*. *infestans* but other data show that UV irradiation or fungal adaptation on sub-lethal doses could mutate oomycete plant pathogens such as *P*. *capsici* [2, 12, 15] and *P*. *nicotianae* [16] for resistance to oxathiapiprolin.

One common practice to delay the buildup of fungicide-resistant sub-populations of a pathogen in the field is to use the at-risk fungicide in a mixture with another fungicide(s) having an unrelated mode of action [17]. Here we measured the efficacy of five solo fungicides (OXPT, CHT, AZ, MPD, and MFX) and four dual mixtures (OXPT+CHT 406SC, OXPT+AZ 170SC, OXPT+MPD 280SC, and OXPT+MFX 280DC) thereof in controlling *P*. *infestans* in tomato in growth chambers and the field. Oxathiapiprolin mixtures aimed to reduce the amount of oxathiapiprolin deployed in the field.Lowering the doses might reduce the selection pressure imposed on the pathogen and lower the risk of resistance buildup.

Our data show that OXPT performed much better per unit weight ai than its partner fungicides. Of the four mixtures, OXPT+MFX usually provided the best disease control, often exhibiting synergistic interaction between its components. This mixture provided excellent curative control of the disease, better than OXPT. It translocated systemically from the bottom treated leaves to the upper untreated leaves and fully protected them from the blight (Fig 8). These enhanced activities might be attributed to its soluble concentrate (SL) formulation, which probably improves its uptake and translocation, and/or to the synergy between its components. Indeed, the data show that OXPT+BF, a mixture of oxathiapiprolin with the blind formulation of MFX, performed significantly better than OXPT in curative treatments. The finding that OXPT+MFX was highly effective against MFX-insensitive isolates of *P*. *infestans* encourages its use in areas in which such isolates dominate. This study showed high efficacy of OXPT in controlling late blight in tomato under field conditions while the other solo fungicides showing moderate control of the disease. In spite of the fact that epidemics were induced by MFX-insensitive isolates, OXPT+MFX performed effectively, better than the other mixtures, reaching the same or better level of control as oxathiapiprolin solo. This suggests that this mixture may be useful against late blight in the field even under pressure of MFX-insensitive isolates of *P*. *infestans*.

We currently attempt to produce OXPT-resistant mutants of *P*. *infestans* in the laboratory by employing the methods we used before to produce mutants resistant to MPD and MFX [21–23]. Testing the usefulness of the above OXPT mixtures against OXPT-resistant mutants will be our next challenge.
